# Decrease in treatment intensity predicts worse outcome in patients with locally advanced head and neck squamous cell carcinoma undergoing radiochemotherapy

**DOI:** 10.1007/s12094-020-02447-y

**Published:** 2020-07-15

**Authors:** S. Mollnar, P. Pondorfer, A.-K. Kasparek, S. Reinisch, F. Moik, M. Stotz, M. Halm, J. Szkandera, A. Terbuch, F. Eisner, A. Gerger, K. S. Kapp, R. Partl, S. Vasicek, T. Weiland, M. Pichler, H. Stöger, D. Thurnher, F. Posch

**Affiliations:** 1 Division of Oncology, Department of Internal Medicine, Comprehensive Cancer Center Graz, Medical University of Graz, Auenbruggerplatz 15, 8036 Graz, Austria; 2 Division of General Otorhinolaryngology, Head and Neck Surgery, Department of Otorhinolaryngology; Comprehensive Cancer Center Graz, Medical University of Graz, Graz, Austria; 3 Center for Biomarker Research in Medicine (CBmed), Graz, Austria; 4 Department of Therapeutic Radiology and Oncology, Comprehensive Cancer Center Graz, Medical University of Graz, Graz, Austria; 5 Research Unit “Non-Coding RNAs and Genome Editing in Cancer”, Medical University of Graz, Graz, Austria; 6 Department of Experimental Therapeutics, MD Anderson Cancer Center, Houston, TX USA

**Keywords:** HNSCC, Radiochemotherapy, Toxicity, Treatment modification

## Abstract

**Purpose:**

Radiochemotherapy (RCT) is an effective standard therapy for locally advanced head and neck squamous cell carcinoma (LA-HNSCC). Nonetheless, toxicity is common, with patients often requiring dose modifications.

**Methods:**

To investigate associations of RCT toxicities according to CTCAE version 5.0 and subsequent therapy modifications with short- and long-term treatment outcomes, we studied all 193 patients with HNSCC who received RCT (70 Gy + platinum agent) at an academic center between 03/2010 and 04/2018.

**Results:**

During RCT, 77 (41%, 95% CI 34–49) patients developed at least one ≥ grade 3 toxicity, including seven grade 4 and 3 fatal grade 5 toxicities. The most frequent any-grade toxicities were xerostomia (*n* = 187), stomatitis (*n* = 181), dermatitis (*n* = 174), and leucopenia (*n* = 98). Eleven patients (6%) had their radiotherapy schedule modified (mean radiotherapy dose reduction = 12 Gy), and 120 patients (64%) had chemotherapy modifications (permanent discontinuation: *n* = 67, pause: *n* = 34, dose reduction: *n* = 7, change to other chemotherapy: *n* = 10). Objective response rates to RCT were 55% and 88% in patients with and without radiotherapy modifications (*p* = 0.003), and 84% and 88% in patients with and without chemotherapy modifications (*p* = 0.468), respectively. Five-year progression-free survival estimates were 20% and 50% in patients with and without radiotherapy modifications (*p* = < 0.001), and 53% and 40% in patients with and without chemotherapy modifications (*p* = 0.88), respectively.

**Conclusions:**

Reductions of radiotherapy dose were associated with impaired long-term outcomes, whereas reductions in chemotherapy intensity were not. This suggests that toxicities during RCT should be primarily managed by modifying chemotherapy rather than radiotherapy.

**Electronic supplementary material:**

The online version of this article (10.1007/s12094-020-02447-y) contains supplementary material, which is available to authorized users.

## Introduction

Platinum-based concomitant radiochemotherapy (RCT) is the current standard of care for locally advanced head and neck squamous cell carcinoma (LA-HNSCC) [1]. A more intensified treatment approach is to administer an induction chemotherapy (ICT) prior to RCT, although this comes at the cost of increased toxicity [2]. Randomized controlled trials have shown that ICT leads to an increased risk of hematologic toxicity, especially neutropenia and leucopenia, with prolonged neutropenia often being responsible for treatment-associated delays [3, 4]. Nonetheless, randomized data suggest favorable effects of ICT on distant metastatic risk and organ preservation rates, but whether ICT + RCT consistently prolongs progression-free (PFS) and overall survival (OS) as compared to RCT alone is still debated [5, 6]. Hence, optimal treatment indication for LA-HNSCC poses a clinical challenge for treating physicians who have to weigh potential oncologic benefits against increased toxicity. It can thus be hypothesized that a better understanding of acute ICT and RCT toxicities including their frequency, predictors and impact on long-term treatment outcome may allow physicians a refined treatment indication in this setting.

The reported frequency of severe treatment-related toxicities during RCT is high, and modifications to the planned RCT schedule such as dose reductions and treatment interruptions are often required [7, 8]. For example, during the last years others have shown that up to 100% of the HNSCC patients undergoing RCT suffer from xerostomia, oral mucositis is reported in more than 95% of patients and some grade of dysphagia occurs in almost two-thirds of patients treated with RCT. Similar figures can be found for the occurrence of dysgeusia. The acute complications also affect nutritional intake, causing malnutrition and severe weight loss consequently [9]. This is again a challenging scenario for treating physicians, who have to decide whether to modify radiotherapy, chemotherapy or both. At present, it is unclear whether these modifications are associated with worse outcome, and whether either modifying radiotherapy or chemotherapy or both is a more favorable approach for limiting potential detriments in oncologic outcome.

In this study, we thus analyzed the association of treatment modifications and short-/long-term oncologic outcome in patients with LA-HNSCC undergoing ICT + RCT or RCT alone.

## Methods

### Study design and population

In this retrospective study, we included all patients with LA-HNSCC who received definitive RCT with or without prior induction treatment at a single academic center (*n* = 193). This study population was selected from the population of patients who were discussed by the local head and neck tumor board between May 2010 and April 2018 (*n* = 1580, Supplementary Fig. 1). Data were retrieved from the center’s electronic health record system as previously described [10, 11], and compiled with an electronic data capture system (“REDCAP”) [12]. Tumors were classified according to the Seventh Edition of AJCC Cancer Staging Manual [13]. All toxicities were scored retrospectively according to Common Toxicity Criteria for Adverse Events (CTC AE v5.0) [14]. Importantly, we only considered toxicities occurring during ICT and/or RCT, but not during long-term oncologic follow-up after completed therapy. The study was approved by the Center’s Ethics Committee (EK-Nr.: 31–091 ex 18/19).

### Radiotherapy treatment

The standard RT protocol at the institution consisted of intensity-modulated radiation therapy (IMRT) using step-and-shoot technique or volumetric-modulated arc therapy (VMAT). The protocol also includes parotid sparing for all patients, except in those were the treating radiation oncologists deemed it to be oncologically inappropriate. The prescribed radiation doses were 70 Gy to high-risk planning target volumes (PTV1), 66–70 Gy to cervical lymph node-positive cases (PTV2), and 50 Gy to prophylactic irradiation areas (PTV3).

### End points

Primary end point of the ICT analysis was the risk of any ≥ G3 toxicity. Secondary end point was the risk of not starting RCT anymore due to toxicity from prior ICT. Tertiary end point was the probability of receiving ICT as scheduled. Exploratory end points were the associations between any ≥ G3 toxicity and (1) objective response rate (ORR) to ICT, (2) the risk of not receiving RCT anymore, and (3) any ≥ G3 toxicity during RCT.

Primary end point of the RCT analysis were the association of RCT modifications and objective response rate and long-term oncologic outcome (PFS + OS). Secondary end points were (1) the risk of any ≥ G3 toxicity, (2) the probability of receiving RCT as planned, (3) pre-specified qualitative analysis of treatment modifications for those patients who did not receive RCT as planned. Tertiary end points were the associations between (1) receiving ICT and developing at least one ≥ G3 toxicity during RCT, and (2) any ≥ G3 toxicity during RCT and ORR to RCT, PFS, OS, and the risks of local progression and distant metastasis. We pre-specified a quantitative and qualitative analysis for the following toxicities: nephrotoxicity, unplanned hospitalization and ototoxicity.

### Statistical methods

All statistical analyses were performed using Stata 15.0 (Stata Corp., Houston, TX, USA). Continuous data were reported as medians [25th–75th percentile], and count data as absolute frequencies (%). Associations between continuous and/or categorical variables were analyzed descriptively with charts and cross-tabulations, and inferentially with Fisher’s exact tests, *χ*2-tests, Wilcoxon’s rank-sum tests, linear regression, as well as uni- and multivariable logistic regression models, respectively. Uni- and multivariable modeling of binary responses was performed with logistic regression. Progression-free (PFS) and overall survival (OS) were estimated with 1-Kaplan–Meier estimators, and risks of local progression, local metastasis and distant metastasis with competing risk cumulative incidence estimators, respectively. Corresponding hazard ratios were modelled with uni- and multivariable Cox regression. Kidney function data over time were analyzed with linear mixed models [15].

## Results

### Cohort description

One-hundred and ninety-three patients were included (Table 1). Most of the patients presented with locally advanced disease [clinical stage III–IV, *n* = 167 (87%)]. Thirty-two (17%) of the patients presented with TNM T1-2 and only 1 (0.5%) and 7 (4%) patients were diagnosed with stage I and stage II HNSCC at initial presentation, respectively. Locoregional lymph node metastases at the neck (TNM N1-3) were present in 149 patients (77%).

**Table 1:** Baseline characteristics of the study population (*n* = 193)

Variable	*n* (% miss.)	Summary measure
**Demographics**		
Age at treatment initiation (years)	193 (0%)	59 [53–66]
Female Gender	193 (0%)	44 (23%)
Body mass index (kg/m^2^)	193 (0%)	24.5 [21.6–27.4]
Never smoked	189 (2%)	40 (21%)
History of alcohol abuse	186 (4%)	86 (46%)
Charleson Comorbidity Index	193 (0%)	4 [4–6]
ECOG^a^ 1 +	193 (0%)	53 (27%)
History or current second primary malignancy	191 (1%)	32 (17%)
Caucasian ethnicity	193 (0%)	193 (100%)
**Tumor characteristics**		
*Tumor location*	193 (0%)	/
Oral cavity	/	22 (11%)
Oropharynx	/	96 (50%)
Hypopharnyx	/	39 (20%)
Larynx	/	23 (12%)
Two-level tumor/others	/	13 (7%)
*Tumor Node Metastasis T*	193 (0%)	/
T1-2	/	32 (17%)
T3	/	57 (30%)
T4a	/	94 (49%)
T4b	/	9 (5%)
TX	/	1 (1%)
*Tumor Node Metastasis N*	193 (0%)	/
N0	/	41 (21%)
N1	/	29 (15%)
N2-N3	/	120 (62%)
NX	/	3 (2%)
*Clinical Stage IV*	193 (0%)	154 (80%)
IVa	/	136 (70%)
IVb	/	18 (9%)
*Human Papilloma Virus status*	193 (0%)	/
p16 (HPV protein 16 kDa) positive	/	39 (20%)
p16 negative	/	68 (35%)
p16 not determined	/	86 (45%)
Tumor grade G3–G4	193 (0%)	104 (53%)

^a^Eastern Cooperative Oncology Group performance status

Seventy-four of the 193 patients (38%) received ICT prior RCT. On average, ICT + RCT patients were younger, had a lower Charlson Comorbidity Index, and a higher ECOG performance status than patients who were treated with RCT alone. Otherwise, gender, smoking and alcohol abuse, as well as primary tumor size and lymph node involvement were comparable between the two groups (Supplementary Table 1). The most frequent ICT regimen was docetaxel, cisplatin, and 5-fluorouracil [TPF, *n* = 62 (84%)], and the ORR to ICT was 43% (95% CI 32–55, Table 2) with only one patient developing progressive disease during ICT. In RCT, according to our local RT protocol, the median projected radiotherapy dose to PTV1 was 70 Gy [25th–75th percentile 70–70, mean 69.9, range 50–70], and the most frequent projected chemotherapy regimen for RCT was cisplatin 100 mg/m^2^ body surface area (BSA) for three cycles (Table 3). The ORR to RCT was 86% (95% CI 80–90, Table 3).

**Table 2:** Characteristics of induction chemotherapy (*n* = 74)

Variable	*n* (% miss.)	Summary measure
**Induction chemotherapy regimens**	74 (0%)	/
Docetaxel/Cisplatin/5-Fluorouracil (“TPF”)	/	62 (84%)
Docetaxel/Cisplatin	/	6 (8%)
Docetaxel/Carboplatin AUC^a^ 5/5-Fluorouracil	/	4 (5%)
Docetaxel/Carboplatin AUC^a^ 5	/	2 (3%)
**Induction chemotherapy responses**	74 (0%)	/
Complete remission (CR)	/	7 (9%)
Partial remission (PR)	/	25 (34%)
Stable disease (SD)	/	12 (16%)
Progressive disease (PD)	/	1 (1%)
Not evaluated (NE)	/	29 (39%)
Objective response rate (ORR = CR + PR)	/	43% (95% CI 32–55)
Disease control rate (DCR = ORR + SD)	/	59% (95% CI 47–71)
**Treatment intensity**		
Received all 3 planned cycles in expected time frame	74 (0%)	50 (68%)
Premature permanent discontinuation	/	8 (11%)
Treatment interruption/Pause	/	6 (8%)
Dose reduction	/	8 (11%)
Change to other therapy	/	2 (3%)
**Treatment toxicity**		
≥ 1 G5 toxicity		0 (0%)
≥ 1 G4 toxicity (Gastric perforation)		1 (1%)
≥ 1 G3 toxicity		16 (22%)
Did not receive CRT anymore		6 (8%)
**Most frequent G3 toxicities**		
Cytopenia		5 (7%)
Infection		5 (7%)
Diarrhea		5 (7%)

^a^Area under the curve

**Table 3:** Characteristics of radiochemotherapy (*n* = 187)

Variable	*n* (% miss.)	Summary measure
**Radiotherapy modality and intensity**		
Projected total radiotherapy dose (Gray)	187 (0%)	70 [70–70]
Administered total radiotherapy dose (Gray)	187 (0%)	70 [70–70]
Did not reach projected total radiotherapy dose	187 (0%)	11 (6%)
Difference between projected and administered total radiotherapy dose (Gray)	11 (0%)	12 [2–46]
**Projected chemotherapy modality**	187 (0%)	/
Cisplatin 100 mg/m^2^ BSA^a^ for 3 cycles	/	141 (75%)
Cisplatin < 100 mg/m^2^ BSA^a^ for 3 cycles	/	8 (4%)
Carboplatin AUC^b^ 2	/	24 (13%)
Carboplatin AUC^b^ 1.5	/	1 (< 1%)
“CALAIS” regimen	/	13 (7%)
**Chemotherapy intensity and main type of modification**	187 (0%)	/
Received all 3 planned cycles in expected time frame	/	67 (36%)
Premature permanent discontinuation	/	69 (37%)
Treatment interruption/Pause	/	34 (18%)
Dose reduction	/	7 (4%)
Change to other therapy	/	10 (5%)
**RCT responses**	187 (0%)	/
Complete remission (CR)	/	132 (71%)
Partial remission (PR)	/	28 (15%)
Stable disease (SD)	/	7 (4%)
Progressive disease (PD)	/	4 (2%)
Not evaluated (NE)	/	16 (9%)
Objective response rate (ORR = CR + PR)	/	86% (95% CI 80–90)
Disease control rate (DCR = ORR + SD)	/	89% (95% CI 84–93)
**Treatment toxicities Grade3-Grade5 (G)**		
Any ≥ G3 toxicity	187 (0%)	77 (41%)
Number of ≥ G3 toxicities per patient	187 (0%)	1 [1–2]
G4 toxicity	187 (0%)	6 (3%)
Mucositis	/	2 (1%)
Dermatitis	/	1 (< 1%)
Sepsis (*Candida* spp.)	/	1 (< 1%)
Gastric perforation	/	1 (< 1%)
Esophageal rupture	/	1 (< 1%)
Nephrotoxicity	/	1 (< 1%)
G5 toxicity	187 (0%)	3 (2%)
Epidural abscess	/	1 (< 1%)
Stroke (*A. cerebri* media)	/	1 (< 1%)
Uncontrollable tumor bleed	/	1 (< 1%)
**Five most frequent any-grade toxicities**	/	/
Xerostomia	/	187 (100%)
Stomatitis	/	181 (97%)
Dermatitis	/	174 (93%)
Leucopenia/Neutropenia	/	98 (52%)
Anemia	/	48 (26%)

^a^Body surface area

^b^Area under the curve

During a median follow-up of 3.6 years for PFS and 4.3 years for OS, 83 (43%) patients died and 65 (34%) patients developed progressive disease, including 40 local recurrences, 15 local metastases and 28 distant metastatic diseases. Five-year OS and PFS estimates were 49% and 46%, and the corresponding 5-year local progression and distant metastasis rates were 35% and 19%, respectively (Supplementary Table 2, Supplementary Fig. 2).

### Toxicities of induction chemotherapy (ICT)

During ICT, 16 of the 74 patients (22%, 95% CI 13–33) experienced a G3 toxicity which was defined as the primary end point. One G4 toxicity was observed (peritonitis after gastric perforation in association with a percutaneous endoscopic gastrostomy tube), and none of the 74 patients developed a G5 toxicity. The most common G3 toxicities were cytopenia (*n* = 5), infection (*n* = 5) and diarrhea (*n* = 5), respectively (Table 2). Other ≥ G3 toxicities observed during ICT included stomatitis (*n* = 3), venous thromboembolism (VTE) (*n* = 1, axillary and subclavian vein), liver toxicity (*n* = 1) and febrile neutropenia (*n* = 2), with one FN occurring after cycle 2 (FN duration: 3 days, hospitalization for 4 weeks for reasons other than FN) and the other FN occurring after cycle 3 (FN duration: 2 days, hospitalization for 3 days). In total, 26 toxicities ≥ G3 were observed, and the median number of ≥ G3 toxicities per patient who developed at least one ≥ G3 toxicity was 1 [1–2, range 1–3]. Any-grade VTE was diagnosed in five patients (7% VTE risk), including one incidental and four symptomatic events. All VTE events were associated with central venous catheters and occurred in the upper extremity or central veins. No case of pulmonary embolism was observed. Three ototoxic events (all hearing impairment) were recorded, with all of these events occurring after the second cycle and one event being associated with additional tinnitus.

Six of the 74 patients (8%, 95% CI 3–17) could not go on to receive further RCT which was defined as the secondary end point. The reasons for not receiving RCT anymore are reported in Supplementary Table 3.

Fifty (68%, 95% CI 56–78) of the 74 patients were able to receive all three cycles of ICT within the planned time interval. Among the 24 patients who did not receive ICT as planned, we observed unplanned permanent ICT discontinuation in 8 cases, treatment interruption/pause in 10 cases, dose reduction in 8 cases, and a change to other therapy in 2 cases (Table 2). Reasons for these deviations from planned ICT and their management are reported in Supplementary Table 4.

Experiencing any ≥ G3 toxicity was not associated with worse ORR during ICT. In detail, ORR was 44% (95% CI 20–70) in patients who experienced at least one ≥ G3 toxicity during ICT, and 43% (95% CI 30–57) in patients who did not experience such toxicity, respectively [odds ratio (OR) = 0.97, 95% CI 0.32–2.97, *p* = 0.963]. The probability of not receiving RCT anymore after ICT was similar between patients with and without any ≥ G3 toxicity. In detail, 2 (13%) and 4 (7%) patients did not receive RCT among the 16 patients with at least one ≥ G3 toxicity and the 58 patients without such toxicity, respectively (Fisher’s exact *p* = 0.604, OR = 1.93, 95% CI 0.32–11.62, *p* = 0.474). Finally, any ≥ G3 toxicity during ICT did also not predict the occurrence of any ≥ G3 toxicity during RCT (OR = 0.69, 95% CI 0.21–2.35, *p* = 0.557).

### Modification of radiochemotherapy (RCT)

Sixty-five (35%, 95% CI 28–42) of the 187 patients who underwent RCT received treatment as planned. The remaining 122 patients either had modifications to radiotherapy (*n* = 2, 1%), chemotherapy (*n *= 111, 59%), or both (*n* = 9, 5%). In the 11 patients with modifications to radiotherapy, the median administered radiotherapy dose to PTV1 was 58 Gy [25th–75th percentile: 11–58, mean 49, range 10–68], and the difference between the projected and eventually administered radiation dose to PTV1 was 12 G [2–46]. Reasons for not reaching the projected radiotherapy dose were patient wish/incompliance (*n* = 5), complications resulting in death (*n* = 3), anasarca (n = 1), gastric perforation with postoperative complications (*n* = 1), and intractable muco-dermatitis (*n* = 1). Among chemotherapy modifications, patients were more often managed by treatment interruption/pause than by dose reduction or change to other therapy, but the risk of premature permanent chemotherapy discontinuation was high (Table 3).

**Fig. 1:**
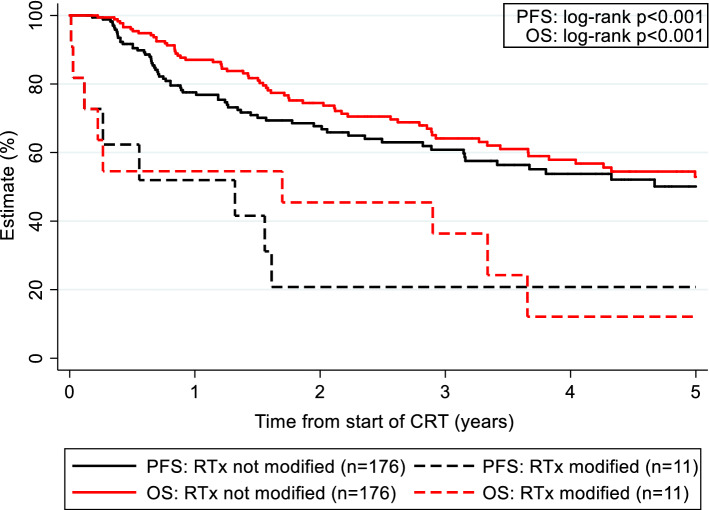
Kaplan–Meier Progression-free (PFS) and Overall Survival (OS) estimates according to concomitant radiotherapy schedule modifications. *RTx* Radiotherapy. Five-year PFS estimates from start of RCT were 20% and 50% in patients with and without radiotherapy modifications (*p* < 0.001) and 53% and 40% in patients with and without chemotherapy modifications (*p* = 0.88). Corresponding 5-year OS estimates were 12% and 53% in patients with and without radiotherapy modifications (*p* < 0.001), and 47% and 54% in patients with and without chemotherapy modifications (*p* = 0.19), respectively

Objective response rates to RCT were 55% and 88% in patients with and without radiotherapy modifications (*p* = 0.003), and 84% and 88% in patients with and without chemotherapy modifications (*p* = 0.468), respectively. Five-year PFS estimates from start of RCT were 20% and 50% in patients with and without radiotherapy modifications (*p* < 0.001, Fig. 1), and 53% and 40% in patients with and without chemotherapy modifications (*p* = 0.88). The corresponding 5-year OS estimates were 12% and 53% in patients with and without radiotherapy modifications (*p* < 0.001, Fig. 1), and 47% and 54% in patients with and without chemotherapy modifications (*p* = 0.19), respectively.

### Toxicities of RCT

Among the 187 patients who underwent RCT, 77 patients (41%, 95% CI 34–49) developed at least one ≥ G3 toxicity. Three toxicities G5 and seven toxicities G4 were observed. G5 toxicities included an epidural abscess, a massive tumor bleeding and a middle cerebral artery infarction. The most frequent toxicities of any grade were xerostomia, stomatitis and dermatitis (Table 3). The vast majority of patients (97%) experienced any-grade stomatitis at any point. Two cases of G4 stomatitis were reported and 29 (16%) patients developed G3 stomatitis. In total, 123 toxicities ≥ G3 were observed, and the median number of ≥ G3 toxicities per patient who developed at least one ≥ G3 toxicity was 1 [1–2, range 1–3].

Patients who received ICT + RCT did not have a higher risk of developing any ≥ G3 toxicity during RCT than patients who were treated with RCT alone (43% vs. 40%, *p* = 0.757).

Importantly, patients who developed at least one ≥ G3 toxicity during RCT had significantly worse short- and long-term outcomes than patients without any ≥ G3 toxicity during RCT, including worse RCT ORRs (75% vs. 93%, *p* = 0.001) and worse 5-year PFS (41% vs. 53%, *p* = 0.040). Five-year risks of death from any cause (56% vs. 45%, *p* = 0.140) and local progression (38% vs. 29%, *p* = 0.075) were numerically, but not statistically significantly worse in patients with at least one ≥ G3 toxicity (44% vs. 55%, *p* = 0.140). Five-year distant metastasis risk was comparable between these two patient groups (17% vs. 20%, p = 0.492).

In univariable regression analysis, the occurrence of at least one ≥ G3 toxicity during RCT predicted worse RCT ORR [univariable odds ratio (OR) = 0.24, 95%CI: 0.10–0.58, *p* = 0.002] and worse 5-year PFS [univariable hazard ratio (HR) = 1.62, 1.02–2.57, *p* = 0.042]. In multivariable analysis adjusting for important confounders and prognostic factors, the adverse associations between at least one ≥ G3 toxicity during RCT and worse RCT ORR prevailed (adjusted OR = 0.26, 95% CI 0.10–0.70, *p* = 0.007), whereas the association with worse 5-year PFS did not (adjusted HR = 1.637, 0.84–2.24, *p* = 0.205, Table 4).

**Table 4:** Two multivariable models for predicting RCT objective response rate and 5-year progression-free survival

Model	Endpoint/estimate type	Variable	Estimate	95% CI (*p*)
#1	Objective response rate (ORR)/Odds Ratio (OR)	≥ 1 G3 toxicity during RCT	0.26	0.10–0.70 (0.007)
		Age (per 5 years increase)	0.70	0.51–0.96 (0.028)
		ECOG^a^ ≥ 1	0.93	0.34–2.56 (0.883)
		Tumor site: Oral cavity	Ref	Ref
		Oropharynx	13.66	3.44–54.25 (0.0001)
		Hypopharnyx	2.96	0.81–10.87 (0.102)
		Larynx	11.00	1.73–69.88 (0.01)
		Two-level tumor/others	5.84	0.91–37.41 (0.063)
#2	5-year progression-free survival (PFS)/Hazard ratio (HR)	≥ 1 G3 toxicity during RCT	1.37	0.84–2.24 (0.205)
		Age (per 5 years increase)	0.93	0.82–1.06 (0.264)
		ECOG^a^ ≥ 1	1.44	0.86–2.39 (0.264)
		Tumor site: Oral cavity	Ref	Ref
		s Oropharynx	0.46	0.22–0.97 (0.040)
		Hypopharnyx	0.63	0.28–1.41 (0.260)
		Larynx	0.52	0.21–1.30 (0.162)
		Two-level tumor/others	0.68	0.26–1.78 (0.429)

^a^Eastern Cooperative Oncology Group performance status

**Fig. 2:**
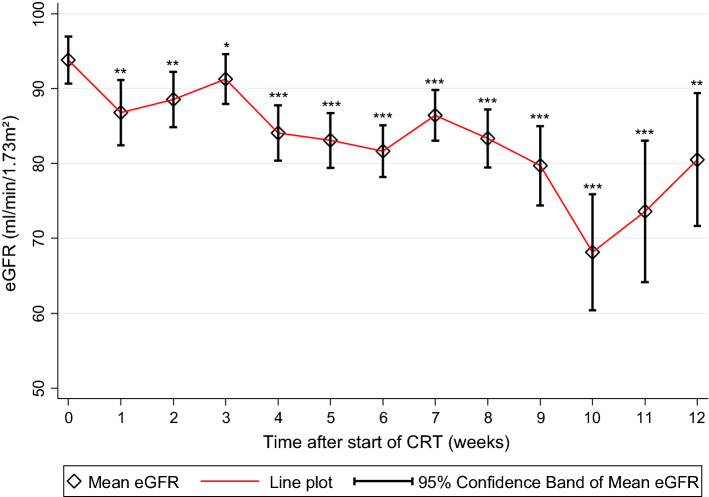
Evolution of kidney function during radiochemotherapy. “Week 0” represents kidney function data immediately before treatment initiation. *p < 0.1, **p < 0.01, ***p < 0.001. *eGFR* Estimated Glomerular Filtration Rate. RCT was associated with a highly significant decline in kidney function. Median eGFR at baseline was 94.7 ml/min/1.73m^2^, 79.1 ml/min/1.73m^2^ after 9 weeks of treatment and did not fully recover over the first 12 weeks after treatment initiation

We pre-specified a quantitative and qualitative analysis for the following RCT toxicities: nephrotoxicity, ototoxicity, and unplanned hospitalization. Overall, RCT was associated with a highly significant decline in kidney function (median eGFR at baseline: 94.7 ml/min/1.73m^2^) and did not fully recover over the first 12 weeks after treatment initiation (*n* = 1542 estimated glomerular filtration rate (eGFR) records, mean records per patient: 8.4, Fig. 2). Nephrotoxicity, as defined by physician adjudication, occurred in 30 patients (16%, 95% CI 11–22). At baseline, median eGFR was similar between patients who did and did not develop nephrotoxicity (95.0 ml/min/1.73m^2^ vs. 94.5 ml/min/1.73m^2^, *p* = 0.782). Importantly, neither higher age (OR per 5 years increase = 0.98, 0.79–1.21, *p* = 0.828), nor ECOG ≥ 1 (OR = 0.93, 0.38–2.23, *p* = 0.863), nor prior ICT (OR = 1.19, 0.53–2.65, *p* = 0.669) predicted a higher risk of nephrotoxicity.

Ototoxicity of any type occurred in 20 patients (11%, 95% CI 7–16), with exact details being reported in the patient matrix of Supplementary Table 5. Interestingly, neither higher age (OR per 5 years increase = 1.02, 0.79–1.33), nor prior ICT (OR = 1.19, 0.46–3.07, *p* = 0.721), nor full-dose RCT treatment without any dose modifications (OR = 0.56, 0.20–1.63, *p* = 0.290) predicted a higher risk of ototoxicity. Moreover, nephrotoxicity during RCT was not associated with ototoxicity (χ^2^ p = 0.248).

Unplanned hospitalizations during RCT occurred in 37 out of 187 patients (20%, 95% CI 15–27). The index reasons for hospitalization are reported in Supplementary Table 6, with poor performance status as the most frequent index reason (*n* = 13). Most hospitalization occurred after the second RCT cycle (*n* = 20). The mean duration of hospital stay was 10 days (25th–75th percentile: 6–12, range 4–42). Importantly, the risk of unplanned hospitalization during RCT was similar in patients who had and had not received prior ICT (20% vs. 19%, *p* = 0.873).

## Discussion

In this retrospective single-center study, we systematically analyzed treatment-related toxicities of ICT and RCT in 193 patients with LA-HNSCC.

In the first part of the analysis, we investigated toxicities during ICT. Here, the risk of developing at least one ≥ G3 toxicity was relatively low. Importantly, only one G4 toxicity and no treatment-related death were observed during ICT. Further, ICT toxicities did not preclude the subsequent administration of RCT. In detail, only 6 of 74 patients did not receive RCT following ICT, and in only 2 of these 6 patients this was due to intractable treatment-related toxicities. Tertiary analyses addressed ICT treatment delivery and showed that a relatively high proportion of patients were able to receive all three ICT cycles within the planned time frame. Furthermore, we observed that toxicities were primarily managed by postponing treatment for a short time period or dose reduction, and only in eight patients by premature permanent ICT discontinuation. Regarding ICT response rates, we did not observe an association between developing any ≥ G3 toxicity and worse ORR. In addition, experiencing any ≥ G3 toxicity was also not associated with the development of any ≥ G3 toxicity during RCT. These findings underline that ICT toxicities (1) are manageable given an adequate patient selection and optimal supportive therapy, (2) do not limit the feasibility of subsequent RCT, and (3) align well with previous studies by Paccagnella et al. and Vermorken et al. [3, 16], and do not support an approach of de-escalating ICT to two instead of three cycles [17].

In the second part of the analysis, we quantified treatment-related toxicities during RCT and their impact on long-term oncologic outcome. Compared to the ICT setting, a much higher proportion of patients experienced at least one ≥ G3 toxicity, including three treatment-related deaths and seven toxicities G4. Furthermore, we noted that patients who experienced at least one ≥ G3 toxicity during RCT had significantly worse short- and long-term outcome. These toxicities eventually led to treatment modifications in two-thirds of patients. Interestingly, we observed that most of these modifications affected chemotherapy and only in 11 patients the total radiation dose was modified. In quantifying the association of these modification with short- and long-term oncologic outcomes, we observed that reductions in concomitant radiotherapy intensity were associated with impaired long-term outcomes, whereas reductions in concomitant chemotherapy intensity were not. In detail, a modified radiation dose predicted significantly lower ORR and 5-year OS and PFS estimates in these patients than in patients without radiotherapy modifications. The third cycle of concomitant chemotherapy was often not administered in the expected time frame or not feasible anymore because of severe hematologic toxicity or muco-dermatitis, a problem which has also been addressed in previous literature [8, 18, 19]. Interestingly, our results do not show any association between chemotherapy modification and long-term treatment outcome. Whether these results are causal cannot be ultimately addressed within our retrospective observational design, but considering that randomized studies on this topic are not feasible, this data may provide at least some guidance to physicians on the most optimal way to modify RCT intensity in a patient with clinically significant toxicity. Specifically, our data suggest that higher-grade toxicities during RCT for locally advanced HNSCC may be better managed by modifying chemotherapy rather than radiotherapy. On the other hand, previous studies have highlighted the importance of reaching a high cumulative cisplatin dose during RCT, especially as this may reduce the risk of distant metastasis [20–22]. A possible reason for the discrepancy between these studies and our data might be the definition of “high cumulative cisplatin dose”, which was defined as receiving more than 200 mg/m^2^ body surface area for the most part [20, 22]. Looking at our study, this specified cutoff value was still reached by the majority of patients who had chemotherapy modification, suggesting that modification of the chemotherapy schedule does not affect short-/long-term outcomes, as long as a relatively high cumulative cisplatin dose, previously defined as > 200 mg/m^2^, is reached.

Finally, several limitations to the study have to be discussed. As with all retrospective studies, selection and/or information bias cannot be ruled out. In terms of information bias, all toxicities were retrospectively adjudicated. This opens up the possibility for misclassifying the severity of toxicities, and/or toxicity underreporting. Another example of potential information bias in our study is the ICT ORR, because more than one-third of the patients who received ICT did not have radiographic staging examinations before continuing with RCT. Thus, our dataset includes a higher proportion of patients with unknown ICT response than what would be expected in a prospective study. Another major limitation of our study is that we did not include data on human papillomavirus (HPV) infection status, because these data were not available for many of our patients at the time of analysis. Furthermore, we included different treatment regimens and dosages in the ICT as well as the RCT group. It is well known that these regimens may differ in their side-effect profile and also their efficacy [23, 24]. Additionally, we did not consider the radiation target volume, which may vary depending on lymph node involvement and tumor size. Next, various HNSCC tumor locations from oral cavity tumors to laryngeal tumors were included. Tumor site is a variable which strongly correlates with overall HNSCC prognosis and outcome rates [25]. Hence, our findings should be interpreted with the necessary caution, as they may not be fully generalizable to each treatment regimen and each HNSCC tumor site. On the other hand, this may also be considered as a strength of the current study, because data may be applicable across the full clinical spectrum of advanced HNSCC therapy. Apart from that, our results on worse PFS in patients having modifications to radiotherapy could be biased by the three cases of death which were also considered in the group of RCT modification, although treatment intensity was altered due to death and not treatment-related toxicity. In this respect, modification to radiotherapy could have been a proxy variable for worse prognosis for reasons not strictly related to radiotherapy, and we may thus have overestimated the adverse association between radiotherapy modifications and oncologic outcomes. Finally, we refrained from an elaborate analysis of two specific long-term RCT toxicities, namely xerostomia and loss of taste, as these long-term toxicities were not the main focus of our study which was concerned with immediate treatment-related toxicities. Apart from that, our study does not provide quantitative assessment of the most common RCT toxicities dysphagia, dermatitis and oral mucositis, as no unified assessment tool was used during data collection. This is a major limitation, because these toxicities can particularly affect the compliance of the patient to treatment and thus our dose-density analyses [26].

We conclude that the frequency of severe ICT toxicities is relatively low in a large real-world cohort of patients with advanced HNSCC, and ICT does not appear to compromise the subsequent delivery of RCT in these patients. Toxicity-related modifications of the radiation dose were found to be associated with impaired oncologic outcome, while this was not evident for chemotherapy modifications. Within the limitations of a retrospective observational study, we thus propose modification of the chemotherapy schedule as a favorable approach to not compromise the delivery of full dose radiotherapy.

## Electronic supplementary material

Below is the link to the electronic supplementary material.

Supplementary file1 (DOCX 144 kb)
