# Supplementary material for: Decrease in treatment intensity predicts worse outcome in patients with locally advanced head and neck squamous cell carcinoma undergoing radiochemotherapy
Source: Clin Transl Oncol. 2020 Jul 15;23(3):543–53. doi: 10.1007/s12094-020-02447-y (PMC7936960; doi:10.1007/s12094-020-02447-y)
Supplement: Supplementary file 1 — Supplementary file1 (DOCX 144 kb) [file 12094_2020_2447_MOESM1_ESM.docx]

# Supplementary Data

**TREATMENT-RELATED REDUCTIONS IN TREATMENT INTENSITY PREDICT WORSE OUTCOME IN PATIENTS WITH LOCALLY-ADVANCED HEAD AND NECK SQUAMOUS CELL CARCINOMA UNDERGOING RADIOCHEMOTHERAPY**

Running Title: Radiochemotherapy toxicities in advanced HNSCC

**Stefanie Mollnar,^1^ Prisca Pondorfer, MD,^2^ Anne-Katrin Kasparek, MD,^1^ Sabine Reinisch, MD,^2^ Florian Moik, MD,^1^ Michael Stotz, MD PhD,^1^ Michael Halm, MD,^1^ Joanna Szkandera, MD,^1^ Angelika Terbuch, MD,^1^ Florian Eisner, MD,^1^ Prof. Armin Gerger, MD MBA,^1,3^ Prof. Karin S. Kapp, MD,^4^ Richard Partl, MD,^4^ Sarah Vasicek, MD,^2^ Thomas Weiland, MD,^2^ Prof. Martin Pichler, MD MSc,^1,5,6^ Prof. Herbert Stöger, MD,^1^ Prof. Dietmar Thurnher, MD^2^ and Florian Posch, MD MSc^1,3^**

^1^Division of Oncology; Department of Internal Medicine; Comprehensive Cancer Center Graz; Medical University of Graz; Graz, Austria

^2^Division of General Otorhinolaryngology, Head and Neck Surgery; Department of Otorhinolaryngology; Comprehensive Cancer Center Graz; Medical University of Graz; Graz, Austria

^3^Center for Biomarker Research in Medicine (CBmed); Graz, Austria

^4^Department of Therapeutic Radiology and Oncology; Comprehensive Cancer Center Graz; Medical University of Graz; Graz, Austria

^5^Research Unit “Non-Coding RNAs and Genome Editing in Cancer”; Medical University of Graz; Graz, Austria

^6^Department of Experimental Therapeutics; MD Anderson Cancer Center; Houston, TX, USA

Address for correspondence:

Florian Posch, MD MSc

Division of Oncology, Department of Internal Medicine

Medical University of Graz

Auenbruggerplatz 15, 8036 Graz – Austria

Tel.: (+43316) 385-31072; Fax: (+43316) 385-13355

E-mail: [florian.posch@medunigraz.at](mailto:florian.posch@medunigraz.at)

Clinical and Translational Oncology

# Supplementary Table 1

| **Variable** | **n**  **(% miss.)** | **RCT**  **(n=119)** | **RCT+ICT (n=74)** | **p** |
| --- | --- | --- | --- | --- |
|  |  |  |  |  |
| **Demographics** |  |  |  |  |
| Age (years) | 193 (0%) | 63 [55-68] | 55 [50-60] | <0.0001 |
| Female Gender | 193 (0%) | 30 (25%) | 14 (19%) | 0.311 |
| BMI (kg/m²) | 193 (0%) | 24.5 [21.3-27.1] | 24.4 [21.9-27.5] | 0.461 |
| Never smoked | 189 (2%) | 27 (23%) | 13 (18%) | 0.456 |
| History of alcohol abuse | 186 (4%) | 57 (50%) | 29 (41%) | 0.247 |
| Charleson Comorbidity Index | 193 (0%) | 5 [4-6] | 4 [3-4] | <0.0001 |
| ECOG 1+ | 193 (0%) | 46 (39%) | 7 (9%) | <0.0001 |
| History or current SPM | 191 (1%) | 25 (21%) | 7 (9%) | 0.032 |
| Caucasian ethnicity | 193 (0%) | 119 (100%) | 74 (100%) | N/A |
|  |  |  |  |  |
| **Tumor characteristics** |  |  |  |  |
| Tumor location | 193 (0%) | / | / | 0.032 |
| ---Oral cavity | / | 15 (13%) | 7 (9%) | / |
| ---Oropharynx | / | 62 (52%) | 34 (46%) | / |
| ---Hypopharnyx | / | 26 (22%) | 13 (18%) | / |
| ---Larynx | / | 7 (6%) | 16 (22%) | / |
| ---Two-level tumor/others | / | 9 (8%) | 4 (5%) | / |
| TNM T4 | 193 (0%) | 62 (52%) | 41 (55%) | 0.655 |
| TNM N2-N3 | 190 (2%) | 71 (61%) | 49 (66%) | 0.485 |
| Clinical Stage IV | 193 (0%) | 93 (78%) | 61 (82%) | 0.471 |
| HPV/p16 positive* | 107 (45%) | 33 (48%) | 6 (16%) | 0.001 |
| Tumor grade G3-G4 | 183 (5%) | 67 (60%) | 37 (51%) | 0.231 |

# Supplementary Table 2

| **Endpoint** | **1-year estimate (95%CI)** | **3-year estimate (95%CI)** | **5-year estimate (95%CI)** |
| --- | --- | --- | --- |
|  |  |  |  |
| Overall survival (OS) | 86% (80-90) | 64% (55-71) | 49% (40-58) |
| Progression-free survival (PFS) | 76% (69-82) | 57% (49-65) | 46% (37-55) |
| Local Progression | 15% (10-21) | 26% (19-33) | 35% (26-43) |
| Distant Metastasis | 6% (3-11) | 17% (11-24) | 19% (13-27) |

# Supplementary Table 3

| **Patient** | **Reason(s) for not receiving RCT anymore** | **Further therapy** |
| --- | --- | --- |
|  |  |  |
| #1 | Gastric perforation during the 1^st^ ICT cycle | Radioimmunotherapy instead of RCT |
| #2 | Docetaxel-associated liver toxicity  during 1^st^ ICT cycle | No more chemotherapy, definitive radiotherapy only |
| #3 | Disease progression after 1^st^ ICT cycle | Further treatment with radioimmunotherapy |
| #4 | Stable disease (SD) after 2^nd^ ICT cycle | Laryngectomy with bilateral neck dissection and postoperative radiotherapy |
| #5 | Low compliance with scheduled treatment visits during ICT, poor response to ICT, worsening performance status, infection during ICT | No more chemotherapy, definitive radiotherapy only |
| #6 | Patient declined RCT after completing ICT | Palliative care |

# Supplementary Table 4

| **Toxicity outcome** | **n (%)** | **Management / Comment** |
| --- | --- | --- |
|  |  |  |
| **Premature permanent ICT discontinuation** | 8 (100%) | / |
| ---Capillary leakage syndrome | 1 (13%) | After cycle 2 |
| ---Gastric perforation | 1 (13%) | During cycle 1 |
| ---Disease progression | 1 (13%) | After cycle 1 |
| ---Liver toxicity | 1 (13%) | After cycle 1 |
| ---Sepsis, Venous Thromboembolism | 1 (13%) | After cycle 2 |
| ---Diarrhea | 1 (13%) | During cycle 3 |
| ---No clinical response | 1 (13%) | After cycle 2 |
| ---Hepatic decompensation | 1 (13%) | After cycle 2 |
|  |  |  |
| **Treatment pause** | 10 (100%) | / |
| ---Venous thrombosis | 1 (10%) | 1 week delay of cycle 3 |
| ---Elevated inflammatory markers | 1 (10%) | 1 week delay of cycle 2 |
| ---MRSA infection | 1 (10%) | 1 week delay of cycle 3 |
| ---Leukocytosis | 1 (10%) | 1 week delay of cycle 2 |
| ---Anemia + nephrotoxicity | 1 (10%) | 1 week delay of cycle 3 |
| ---Port-A-Cath infection | 1 (10%) | 3 weeks delay of cycle 3 |
| ---Mucositis + diarrhea | 1 (10%) | 1 week delay of cycle 2 |
| ---Dental infection + Anemia | 1 (10%) | 1 week delay of cycle 2 |
| ---Leukopenia | 1 (10%) | 1 week delay of cycle 3 |
| ---Infection | 1 (10%) | 1 week delay of cycle 3 |
|  |  |  |
| **Dose reduction** | 8 (100%) | / |
| ---Diarrhea + mucositis | 1 (13%) | No 5-FU during cycles 2 & 3 |
| ---Diarrhea | 1 (13%) | 5-FU reduced to 75% for cycle 3 |
| ---Diarrhea | 1 (13%) | 5-FU reduced to 80% for cycle 2 and to 60% for cycle 3 |
| ---Cytopenia | 1 (13%) | Carboplatin reduced to 90% for cycles 2 & 3 |
| ---Worsening performance status | 1 (13%) | Cisplatin, docetaxel, and 5-FU all reduced to 80% for cycles 2 & 3 |
| ---Pancytopenia | 1 (13%) | Cisplatin, docetaxel, and 5-FU all reduced to 75% for cycle 3 |
| ---Anemia | 1 (13%) | Carboplatin reduced to  AUC4 for cycle 3 |
| ---Mucositis | 1 (13%) | 5-FU reduced to 75% for cycle 3 |
|  |  |  |
| **Change to other treatment** | 2 (100%) | / |
| Nephrotoxicity | 1 (50%) | Change from cisplatin to carboplatin after cycle 1 |
| Nephrotoxicity | 1 (50%) | Change from cisplatin to carboplatin after cycle 2 |

# Supplementary Table 5

| **Patient** | **#1** | **#2** | **#3** | **#4** | **#5** | **#6** | **#7** | **#8** | **#9** | **#10** | **#11** | **#12** | **#13** | **#14** | **#15** | **#16** | **#17** | **#18** | **#19** | **#20** |
| --- | --- | --- | --- | --- | --- | --- | --- | --- | --- | --- | --- | --- | --- | --- | --- | --- | --- | --- | --- | --- |
|  |  |  |  |  |  |  |  |  |  |  |  |  |  |  |  |  |  |  |  |  |
| **Pretherapeutic sensorineural hearing loss** | 1 | × | 0 | 1 | 1 | 3 | 0 | 1 | × | 1 | 1 | 1 | 3 | 1 | 0 | 1 | 0 | 0 | 1 | 1 |
| **Posttherapeutic sensorineural hearing loss** | 1 | × | 1 | 5 | 2 | × | 1 | 1 | 4 | 4 | 1 | 4 | 3 | 2 | 5 | 1 | 1 | 5 | × | 2 |
| **Side of hearing loss**  **1=right, 2=left, 3=bilateral** | 3 | - | 3 | 3 | 3 | 3 | 3 | 3 | 3 | 3 | 3 | 3 | 3 | 1 | 3 | 3 | 1 | 3 | 3 | 3 |
| **Hearing impairment likely due to ototoxic treatment**  **0=no, 1=yes** | 0 | - | 1 | 1 | 1 | - | 1 | 0 | 1 | 1 | 0 | 1 | 0 | 1 | 1 | 0 | 1 | 1 | - | 0 |
| *Classification of sensorineural hearing loss according to severity*  *0=normal, 1=mild, 2=mild-intermediate, 3=intermediate, 4=intermediate-severe, 5=severe*  ×*=no audiometry was performed*  *- = not applicable* | | | | | | | | | | | | | | | | | | | | |

# Supplementary Table 6

| **Reason for unplanned hospitalization** |  | **Frequency** |
| --- | --- | --- |
|  |  |  |
| Poor performance status |  | n=13 |
| Infection |  | n=7 |
| Nausea/Emesis |  | n=6 |
| Cytopenia |  | n=3 |
| Mucositis/Stomatitis |  | n=2 |
| Gastric perforation |  | n=1 |
| Dysphagia |  | n=1 |
| Nephrotoxicity |  | n=1 |
| PEG probe complication |  | n=1 |
| Electrolyte disorder |  | n=1 |
| Unclear |  | n=1 |

# Supplementary Table legends

**Supplementary Table 1. Distribution of baseline characteristics by treatment group (n=193).** n (%miss.) reports the number of fully observed patients (% missing). Data are reported as medians [25^th^-75^th^ percentile] or absolute counts (%). P-values are from rank-sum tests and χ^2^-tests. *p16 status was missing in a large proportion of patients. Abbreviations: RCT – radiochemotherapy, ICT – Induction chemotherapy, BMI – Body mass index, ECOG – Eastern Cooperative Oncology Group, N/A – not applicable, SPM – Second primary malignancy, TNM – Tumor Node Metastasis classification, HPV – Human Papilloma Virus, p16 – HPV protein 16kDa.

**Supplementary Table 2.** **Crude outcome risks of the overall study cohort (n=193).** Overall- and progression-free survival were estimated with Kaplan-Meier estimators, and risk of local progression and distant metastasis with competing risk cumulative incidence estimators according to Marubini & Valsecchi treating death-from-any-cause as the competing event of interest. Abbreviations: 95%CI – 95% confidence interval.

**Supplementary Table 3. Reasons(s) for not receiving RCT anymore (n=6).** Abbreviations: ICT – induction chemotherapy, RCT – radiochemotherapy.

**Supplementary Table 4. Analysis of ICT treatment modification.** Abbreviations: ICT – induction chemotherapy, 5-FU – 5-Fluourouracil, AUC – area under the curve, MRSA – methicillin-resistant Staphylococcus aureus.

**Supplementary Table 5. Patient matrix of ototoxicity.** Overall, 20 patients developed an ototoxic complication. In the table, each of these 20 patients represents a column. Ototoxic complications were adjudicated by a resident in ENT (ear, nose, throat) medicine/surgery (PP).

**Supplementary Table 6. Index reasons for unplanned hospitalization during RCT.**

# Supplementary Figure 1


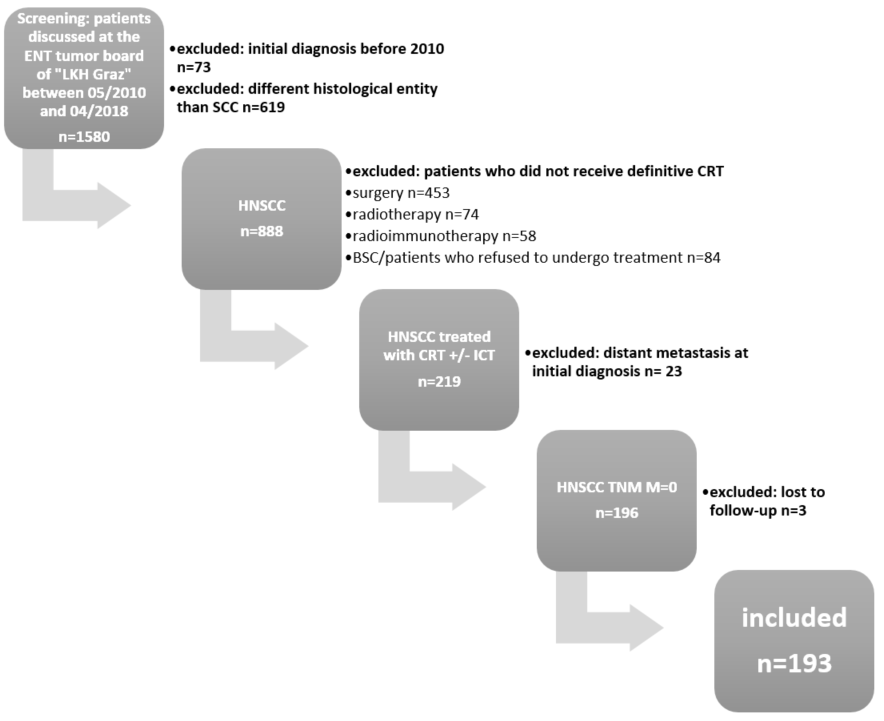


# Supplementary Figure 2

# Supplementary Figure legends

**Supplementary Figure 1. Patient flow - selection of the study population.** Abbreviations: ENT – “ear, nose, throat”, HNSCC – Head and neck squamous cell carcinoma, BSC – Best Supportive Care, TNM – Tumor Node Metastasis classification.

**Supplementary Figure 2.** **Five-year outcomes of the overall study population (n=193).** Overall (OS) and progression-free survival (PFS) were estimated with Kaplan-Meier estimators, whereas risks of local progression and distant metastasis were computed with competing risk cumulative incidence estimators treating death-from-any-cause as the competing event of interest.
